# Carbuncles in Horses

**Published:** 1887-10

**Authors:** Daniel D. Lee

**Affiliations:** Instructor in Anatomy and Operative Veterinary Surgery, Harvard University


### Art. XXXII.—CARBUNCULUS IN HORSES.

BY DANIEL D. LEE, M.D.V.
*Instructor in Anatomy and Operative Veterinary Surgery, Harvard University.*

In Volume IV. of Pepper's "System of Medicine" is the following account of Carbunculus as it appears in man :

"Carbunculus is a firm, more or less circumscribed, painful, deep-seated inflammation of the skin and subcutaneous structures, variable as to size, terminating in a slough. General malaise, slight fever and chilliness precede and usher in the disease. Locally, there appears at first a more or less circumscribed circular redness, with swelling, tenderness and pain. Soon a phlegmonous inflammation develops, the surface at times showing vesiculation, the lesion involving an area several inches in diameter and of considerable depth. The progress of the disease is not uniform. At the end of a week or two suppuration is fully established, the first signs of this process appearing about the hair follicles. The tissues are now soft and boggy ; the skin becomes gangrenous, breaking down at numerous points, disclosing centres of suppuration, giving the lesion a cribiform appearance. Finally the whole mass sloughs away, either as an entirety or in portions, and results in an open, deep ulcer, with hard and raised edges, which gradually granulates and heals, leaving a pigmented cicatrix. The area involved varies, and may be extensive, sometimes as much as six or eight inches in diameter. The favorite localities for its development are the nape of the neck, shoulder, back and buttocks. As a rule, the process ends in three to six weeks. Uusually only one lesion exists. When there are several, or where they follow each other in succession, the general condition is apt to become markedly depressed, and even a fatal result is not at all uncommon. * * * *

The inflammation starts simultaneously at numerous points, usually from the hair follicles, sweat and sebaceous glands, extending in all directions, and usually terminating in gangrene of the whole area. The inflammatory centres break down rapidly, from each of which the collected pus finds its way to the surface, thus producing the cribiform appearance. According to Warren, the pus ascends by way of the columnae adiposae to the hair follicles, and thence to the surface. The process may involve fascia muscles and even periosteum and bone. The disease is to be distinguished from *furnucle* by its greater size and flatness, and the multiple points of suppuration. From erysipelas, to which in the beginning it may have some resemblance, it is to be differentiated by the hardness, painfulness and circumscribed character of the lesion."

So much for the description of carbunculus in the human being; in the horse the symptoms are almost identical, with a few minor exceptions. Williams, in his Surgery, describes, under the head of "Carbuncle of the Coronary Band, or Furunculus," a disease seen in horses, which appeared in this locality last winter as an enzootic, and which was variously called "Foot Rot, "Stinking Scratches," "Suppurative Cellulitis" and "Erysipelas."

Williams' description of this disease is as follows: "It consists of an oval or irregular portion of the coronary substance becoming infiltrated with unhealthy lymph, forming at first a hard swelling of variable size, very painful, sometimes extending all around the coronet, or appearing at other times as several patches, or spots, of inflammation, accompanied by great pain, lameness and high degree of febrile disturbance. After a few days suppuration sets in, most commonly at several points, with ulceration of the softened portions, forming so many apertures or sinuses, which discharge a thin, unhealthy, ichorous pus. The apertures enlarge by ulceration, or even sloughing of their borders, and by confluence constitute a most formidable-looking and unhealthy wound. In aggravated and malignant cases other inflammatory points appear upon the pastern and fetlock, extending, in some instances, as high as the knee or hock, causing sloughing of great patches of skin and subcutaneous tissues, exposing the tendons, nerves, blood vessels, and even the cavities of the joints, and producing so high a state of fever and suffering as to destroy the animal's life."

In the greater number of cases last winter and spring the carbuncle appeared at some distance away from the coronary band, an attack of ordinary scratches generally preceding it, and in all probability this attack of scratches was the primary cause of setting up the inflammation in the hair follicles, sweat and sebaceous glands, which finally resulted in the carbuncle. Three years ago an outbreak of this disease occurred, but it differed from the one we have just had in that the carbuncles were most frequently seated just between hoof and hair and extended downwards and around the coronary band, often causing the loss of the hoof and of course making the constitutional disturbance much greater when that structure was involved. In fact, in all cases in horses, as the carbuncles in these outbreaks were situated on the legs, the constitutional disturbance was much more severe from the fact that weight was borne on the diseased leg, than it would have been if the carbuncle was situated in a less dependent and less used part of the body, as on the neck, or shoulders, or buttocks, where it appears in man. I have seen the disease originate around an interfering cut, the inflammation in the hair follicles, sweat and sebaceous glands being set up in this case by the bruising of the tissues. In all cases which I have personally seen, some lesion like scratches, an interfering cut, or a bruise have always been *primarily* the cause of the carbuncle—the animal, however, always being in a run-down condition. I have, nevertheless, heard of several cases in which the carbuncle appeared on the leg without any apparent cause other than that the animal was overworked and somewhat debilitated. The class of horses attacked are mainly hard-worked truck horses and heavy-built draught horses; light and well-cared-for horses less frequently. When the carbuncle began the sloughing and gangrene appeared sometimes in two days, and always in three or four. In light and well-bred horses the process was generally confined to one spot, and, after the separation of the slough, recovery was rapid ; some being able to work in two days, others in two or three weeks. In heavy and coarse-bred horses the carbuncles were, as a rule, multiple, involving the coronary substance and leg, the sloughing process extending, constitutional disturbance very severe, sometimes causing the death of the animal with all the symptoms of blood-poisoning, and the process involving the tendons, ligaments and fuscia of the part, or the hoof, to such an extent as to render the animal useless and make it necessary to kill it.

The discharge from the ulcers left by the separation of the sloughs is of the most offensive character, and in severe cases is mixed with blood and the remains of the cast-off tissues.

When first called to see the case it may resemble one of ordinary severe scratches, but the pain on manipulation is very intense and parts moist and soft, not hard and "gummy," as in scratches ; as soon as the sloughing and discharge set in all doubt ceases. If the coronary band is involved no difficulty of diagnosis is met with.

*Prognosis.*—Favorable in light, well-bred horses, and in all those where the carbuncle is single and not near the coronary band, recovery in from one to six weeks, accordto the rapidity of the healing of the ulcer. In coarse-bred and heavy horses, the prognosis is always grave, especially if the constitutional disturbance is severe. Their recovery is always slow, and if they once give up and lie down, they don't get up again.

*Treatment.*—Poultices to the part, frequently changed, irrigation with solution of corrosive sublimate, 1–1000. Some anti septic ointment should be spread around the coronet to keep the discharge from adhering. Give as much nutritious food as possible. An ounce of quinine will often work wonders, given well dissolved in water with a little sulphuric acid. It is sometimes necessary to use slings. After the sloughs separate, the ulcers have to be well stimulated with nitrate of silver, before they show any signs of healing.

